# Zn_2_(TeO_3_)Br_2_

**DOI:** 10.1107/S1600536808011252

**Published:** 2008-04-26

**Authors:** Dong Zhang, Mats Johnsson

**Affiliations:** a Inorganic Chemistry, Stockholm University, S-106 91 Stockholm, Sweden

## Abstract

Single crystals of dizinc tellurium dibromide trioxide, Zn_2_(TeO_3_)Br_2_, were synthesized *via* a transport reaction in sealed evacuated silica tubes. The compound has a layered crystal structure in which the building units are [ZnO_4_Br] distorted square pyramids, [ZnO_2_Br_2_] distorted tetra­hedra, and [TeO_3_ *E*] tetra­hedra (*E* being the 5*s* ^2^ lone pair of Te^4+^) joined through sharing of edges and corners to form layers of no net charge. Bromine atoms and tellurium lone pairs protrude from the surfaces of each layer towards adjacent layers. This new compound Zn_2_(TeO_3_)Br_2_ is isostructural with the synthetic compounds Zn_2_(TeO_3_)Cl_2_, CuZn(TeO_3_)_2_, Co_2_(TeO_3_)Br_2_ and the mineral sophiite, Zn_2_(SeO_3_)Cl_2_.

## Related literature

For related literature, see: Becker *et al.* (2006 ▶); Johnsson & Törnroos (2003*a* ▶,*b* ▶, 2007 ▶); Semenova *et al.* (1992 ▶); Brown & Altermatt (1985 ▶); Galy *et al.* (1975 ▶).

## Experimental

### 

#### Crystal data

Zn_2_(TeO_3_)Br_2_
*M* *_r_* = 466.18
Orthorhombic, 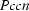

*a* = 10.5446 (2) Å
*b* = 16.0928 (2) Å
*c* = 7.7242 (1) Å
*V* = 1310.74 (3) Å^3^
*Z* = 8
Mo *K*α radiation
μ = 23.79 mm^−1^
*T* = 293 (2) K
0.20 × 0.16 × 0.04 mm

#### Data collection

Oxford Diffraction Xcalibur3diffractometer
Absorption correction: analytical (*CrysAlis RED*; Oxford Diffraction, 2007); *T*_min_ = 0.05, *T*_max_ = 0.35
15561 measured reflections
1290 independent reflections
1201 reflections with *I* > 2σ(*I*)
*R*_int_ = 0.026

#### Refinement

*R*[*F* ^2^ > 2σ(*F* ^2^)] = 0.022
*wR*(*F* ^2^) = 0.055
*S* = 1.09
1290 reflections
74 parameters
Δρ_max_ = 1.08 e Å^−3^
Δρ_min_ = −0.82 e Å^−3^

### 

Data collection: *CrysAlis CCD* (Oxford Diffraction, 2006 ▶); cell refinement: *CrysAlis RED* (Oxford Diffraction, 2006 ▶); data reduction: *CrysAlis RED*; program(s) used to solve structure: *SHELXS97* (Sheldrick, 2008 ▶); program(s) used to refine structure: *SHELXL97* (Sheldrick, 2008 ▶); molecular graphics: *DIAMOND* (Bergerhoff, 1996 ▶); software used to prepare material for publication: *enCIFer* (Allen *et al.*, 2004 ▶).

## Supplementary Material

Crystal structure: contains datablocks I, global. DOI: 10.1107/S1600536808011252/pk2093sup1.cif

Structure factors: contains datablocks I. DOI: 10.1107/S1600536808011252/pk2093Isup2.hkl

Additional supplementary materials:  crystallographic information; 3D view; checkCIF report

## Figures and Tables

**Fig. 1.**
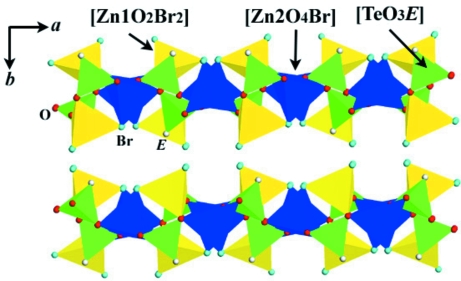
The layer features in Zn2(TeO3)Br2 along [001].

**Fig. 2.**
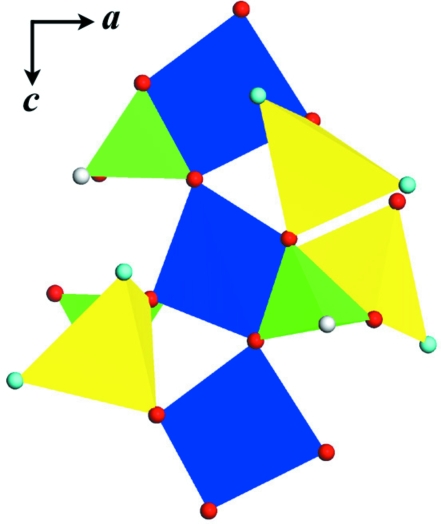
Arrangement of coordination polyhedra around a central [Zn2O4Br] square pyramid. The polyhedra and atom labels are as in Figure 1.

## supplementary crystallographic information

### Comment

The synthesis and crystal structure of the new compound Zn_2_(TeO_3_)Br_2_ is a further result of an ongoing study investigating the rich chemistry of tellurium oxohalides. The tellurium atom has a typical one-sided threefold coordination due to the presence of its lone pair 5 s^2^ (designated E) and the coordination polyhedron is that of a tetrahedron [TeO_3_E].

Zn1 is coordinated by two oxygen atoms and two bromine atoms completing a distorted tetrahedron [Zn1O_2_Br_2_]. Zn2 is coordinated by four oxygen atoms and one bromine atom to complete a distorted square pyramid [Zn2O_4_Br]. A distorted octahedron [Zn2O_4_Br_2_] is formed if Br1 is also taken into account. However, the distance Zn2–Br1 is long [3.3915 (8) Å] and Zn2 is located on the Br2 side of the oxygen plane. Bond valence sum calculations according to Brown & Altermatt (1985) gives a negligible contribution from Br1 suggesting that it should not be considered bonded to Zn2. The three different building units [Zn1O_2_Br_2_], [Zn2O_4_Br] and [TeO_3_E] are connected so that infinite layers are formed, see Figure 1.

Each [Zn2O_4_Br] polyhedron is linked to two other [Zn2O_4_Br] polyhedra by corner sharing so that infinite chains are formed along [001] throughout the layers. Those chains are separated by [Zn1O_2_Br_2_] and [TeO_3_E] groups. Each [Zn2O_4_Br] polyhedron further shares three corners with different [Zn1O_2_Br_2_] groups. The [Zn2O_4_Br_2_] polyhedra also share two corners and one edge with different [TeO_3_E] groups, see Figure 2. The stereochemically active Te lone-pairs are located in the space in between the layers of the structure, pointing towards the space between the likewise protruding Br atoms of the opposite layer. The shortest cation-anion distances between adjacent layers, Zn1–Br1 3.8914 (8) Å, Zn1–Br2 5.3726 (8) Å, Zn2–Br1 4.6898 (8) Å and Te–Br1 3.3904 (6) Å, are similar to or larger than the cation-cation separation within the layers; Zn1^···^Zn1 4.2315 (11) Å, Zn1^···^Zn2 3.3127 (8) Å, Zn2^···^Zn2 3.8755 (1) Å, Te^···^Te 4.4788 (6) Å, Te^···^Zn1 3.4097 (6) Å and Te^···^Zn2 3.0815 (6) Å. This fact indicates the absence of strong contacts between the charge neutral layers and suggests that they are connected only *via* van der Waals interactions, see Figure 1. Each layer can thus be considered as an infinite two-dimensional molecule.

Assuming a Te–E radius of 1.25 Å, which is the average found for Te^4+^–E by Galy *et al.* (1975), the fractional coordinates for the lone-pair E are; *x* = -0.0237, *y* = 0.6565, *z* = 0.1545. This gives contacts E˙˙˙Br1 and E˙˙˙Br2 of ~2.96 and ~2.81 Å, respectively.

The present compound is isostrucural with Zn_2_(TeO_3_)Cl_2_ (Johnsson & Törnroos. 2003*a*), CuZn(TeO_3_)Cl_2_ (Johnsson & Törnroos, 2003*b*) and Co_2_(TeO_3_)Br_2_ (Becker *et al.*, 2006). The mineral Sophiite Zn_2_(SeO_3_)Cl_2_ (Semenova *et al.*, 1992) is also to be considered as isostructural with Zn_2_(TeO_3_)Br_2_, although there is a difference in that the coordination around Zn2 in the mineral can be considered to form a distorted octahedron [Zn2O_4_Cl_2_] with Zn2 located in the oxygen square plane, rather than a square pyramid [Zn2O_4_Br] as in Zn_2_(TeO_3_)Br_2_. Related compounds are Co_2_(TeO_3_)Cl_2_ (Becker *et al. *, 2006) that crystallizes in the monoclinic space group *P*2_1_/*m* and Zn_2_(SeO_3_)Cl_2_ (Johnsson & Törnroos, 2007) a synthetic monoclinic (*P*2_1_/*c*) polymorph of the mineral sophiite.

### Experimental

The synthesis of Zn_2_(TeO_3_)Br_2_ was made by chemical transport reactions in sealed evacuated silica tubes. The compound appeared when searching for new compounds in the system Zn^2+^—O—Br. The starting materials were ZnO (ABCR, +99%), ZnBr_2_ (ABCR, +99%), and TeO_2_ (ABCR, +99%). The preparation of crystals was made from a non stoichiometric mixture of ZnO: ZnBr_2_: TeO_2_ = 1:5:4, which after mixing in a mortar was put into a silica tube (length ~6 cm) which was then evacuated. The tube was heated for 120 h at 830 K in a muffle furnace. The product appeared as colourless transparent plate-like single crystals and powder. The crystals were found to be hygroscopic. The synthesis product was characterized in a scanning electron microscope (SEM, Jeol 7000 F) equipped with an energy-dispersive spectrometer on 4 different single crystals giving a composition of 35.7 ± 2.0 at % Zn, 19.4 ± 0.9 at % Te, 44.1 ± 0.8 at % Br. No significant amount of Si originating from the silica tubes was detected; 0.80 ± 0.5 at% Si.

### Refinement

The maximum residual peak (1.08) is located at 0.82 Å from Te and the largest hole (-0.82) at 0.92 Å from Te.

### Crystal data

**Table:**

Zn_2_(TeO_3_)Br_2_	*F*(000) = 1648
*M**_r_* = 466.18	*D*_x_ = 4.725 Mg m^−^^3^
Orthorhombic, *P**c**c**n*	Mo *K*α radiation, λ = 0.71073 Å
Hall symbol: -P 2ab 2ac	Cell parameters from 13770 reflections
*a* = 10.5446 (2) Å	θ = 3.7–33.2°
*b* = 16.0928 (2) Å	µ = 23.79 mm^−^^1^
*c* = 7.7242 (1) Å	*T* = 293 K
*V* = 1310.74 (3) Å^3^	Block, colourless
*Z* = 8	0.21 × 0.16 × 0.04 mm

### Data collection

**Table:**

Oxford Diffraction Xcalibur3 diffractometer	1290 independent reflections
Radiation source: fine-focus sealed tube	1201 reflections with *I* > 2σ(*I*)
graphite	*R*_int_ = 0.026
φ and ω scans	θ_max_ = 26.3°, θ_min_ = 4.1°
Absorption correction: analytical (*CrysAlis RED*; Oxford Diffraction, 2007)	*h* = −12→12
*T*_min_ = 0.05, *T*_max_ = 0.35	*k* = −20→20
15561 measured reflections	*l* = −9→9

### Refinement

**Table:**

Refinement on *F*^2^	Primary atom site location: structure-invariant direct methods
Least-squares matrix: full	Secondary atom site location: difference Fourier map
*R*[*F*^2^ > 2σ(*F*^2^)] = 0.022	*w* = 1/[σ^2^(*F*_o_^2^) + (0.0333*P*)^2^ + 5.0559*P*] where *P* = (*F*_o_^2^ + 2*F*_c_^2^)/3
*wR*(*F*^2^) = 0.055	(Δ/σ)_max_ = 0.001
*S* = 1.09	Δρ_max_ = 1.08 e Å^−^^3^
1290 reflections	Δρ_min_ = −0.82 e Å^−^^3^
74 parameters	Extinction correction: *SHELXL97* (Sheldrick, 2008), Fc^*^=kFc[1+0.001xFc^2^λ^3^/sin(2θ)]^-1/4^
0 restraints	Extinction coefficient: 0.00342 (15)

### Special details

**Table:**

Experimental. a multifaceted crystal model based on expressions derived by Clark & Reid (1995)]
Geometry. All e.s.d.'s (except the e.s.d. in the dihedral angle between two l.s. planes) are estimated using the full covariance matrix. The cell e.s.d.'s are taken into account individually in the estimation of e.s.d.'s in distances, angles and torsion angles; correlations between e.s.d.'s in cell parameters are only used when they are defined by crystal symmetry. An approximate (isotropic) treatment of cell e.s.d.'s is used for estimating e.s.d.'s involving l.s. planes.
Refinement. Refinement of *F*^2^ against ALL reflections. The weighted *R*-factor *wR* and goodness of fit *S* are based on *F*^2^, conventional *R*-factors *R* are based on *F*, with *F* set to zero for negative *F*^2^. The threshold expression of *F*^2^ > σ(*F*^2^) is used only for calculating *R*-factors(gt) *etc*. and is not relevant to the choice of reflections for refinement. *R*-factors based on *F*^2^ are statistically about twice as large as those based on *F*, and *R*- factors based on ALL data will be even larger.

### Fractional atomic coordinates and isotropic or equivalent isotropic displacement parameters (Å^2^)

**Table:**

	*x*	*y*	*z*	*U*_iso_*/*U*_eq_	
Te	0.02983 (3)	0.592957 (18)	0.21187 (3)	0.01207 (12)	
Zn1	−0.00948 (6)	0.39234 (4)	0.34333 (7)	0.01639 (15)	
Zn2	0.26526 (5)	0.52410 (4)	0.39968 (7)	0.01671 (16)	
Br2	0.20748 (5)	0.37261 (3)	0.43887 (7)	0.02286 (16)	
Br1	−0.08838 (7)	0.29014 (3)	0.15586 (7)	0.03163 (18)	
O2	0.1882 (3)	0.5524 (2)	0.1440 (4)	0.0160 (7)	
O1	−0.0568 (3)	0.4903 (2)	0.1969 (4)	0.0155 (7)	
O3	0.0906 (3)	0.5786 (2)	0.4378 (4)	0.0158 (7)	

### Atomic displacement parameters (Å^2^)

**Table:**

	*U*^11^	*U*^22^	*U*^33^	*U*^12^	*U*^13^	*U*^23^
Te	0.0105 (2)	0.01301 (18)	0.01267 (17)	0.00006 (11)	−0.00162 (10)	0.00168 (10)
Zn1	0.0188 (3)	0.0174 (3)	0.0130 (3)	0.0018 (2)	0.0002 (2)	−0.0011 (2)
Zn2	0.0110 (3)	0.0260 (3)	0.0132 (3)	0.0033 (2)	−0.0005 (2)	−0.0016 (2)
Br2	0.0163 (3)	0.0204 (3)	0.0318 (3)	0.0013 (2)	−0.00128 (19)	0.00377 (19)
Br1	0.0530 (4)	0.0191 (3)	0.0227 (3)	−0.0070 (3)	−0.0101 (2)	−0.0017 (2)
O2	0.0094 (17)	0.0266 (19)	0.0120 (14)	0.0012 (14)	0.0001 (12)	−0.0007 (13)
O1	0.0125 (18)	0.0165 (16)	0.0174 (15)	−0.0034 (14)	−0.0037 (13)	0.0013 (13)
O3	0.0138 (18)	0.0230 (18)	0.0105 (15)	0.0024 (15)	−0.0023 (12)	−0.0016 (12)

### Geometric parameters (Å, °)

**Table:**

Te—O2	1.867 (3)	Zn1—Br2	2.4247 (8)
Te—O3	1.873 (3)	Zn2—O2^ii^	2.002 (3)
Te—O1	1.891 (3)	Zn2—O1^iii^	2.033 (3)
Zn1—O3^i^	1.952 (3)	Zn2—O3	2.061 (3)
Zn1—O1	2.004 (3)	Zn2—O2	2.184 (3)
Zn1—Br1	2.3438 (8)	Zn2—Br2	2.5310 (8)
			
O2—Te—O3	85.00 (14)	O2^ii^—Zn2—O3	89.30 (13)
O2—Te—O1	96.31 (15)	O1^iii^—Zn2—O3	157.82 (14)
O3—Te—O1	96.55 (14)	O2^ii^—Zn2—O2	153.62 (18)
O3^i^—Zn1—O1	101.01 (14)	O1^iii^—Zn2—O2	92.03 (12)
O3^i^—Zn1—Br1	123.23 (11)	O3—Zn2—O2	73.00 (12)
O1—Zn1—Br1	96.62 (10)	O2^ii^—Zn2—Br2	99.54 (10)
O3^i^—Zn1—Br2	100.43 (10)	O1^iii^—Zn2—Br2	98.97 (10)
O1—Zn1—Br2	120.65 (10)	O3—Zn2—Br2	100.22 (10)
Br1—Zn1—Br2	115.54 (3)	O2—Zn2—Br2	102.69 (10)
O2^ii^—Zn2—O1^iii^	98.37 (13)		

Symmetry codes: (i) −*x*, −*y*+1, −*z*+1; (ii) −*x*+1/2, *y*, *z*+1/2; (iii) *x*+1/2, −*y*+1, −*z*+1/2.
